# HAVING A SENSE OF PURPOSE IN LIFE AS A PROTECTIVE FACTOR FOR COGNITIVE DECLINE IN LATER ADULTHOOD

**DOI:** 10.1093/geroni/igad104.1025

**Published:** 2023-12-21

**Authors:** Giyeon Kim

**Affiliations:** Chung-Ang University, Seoul, Republic of Korea

## Abstract

The purpose of this symposium presentation is to discuss the role of having a sense of purpose in life in protecting against cognitive decline in later adulthood. The speaker presents recent findings on purpose in life as a protective factor for cognitive decline from a nationally representative sample of older adults. First, the speaker presents evidence that older adults with a purpose in life showed slower trajectories of cognitive decline during the 6-year period and that purpose in life was a significant moderator of age- and race/ethnicity-cognitive decline associations in later adulthood. Second, the speaker presents evidence that a significant moderating role of purpose in life in the relation of widowhood to total cognition and fluid intelligence, but not in the widowhood-crystallized intelligence relation and that the significant moderating role of purpose in life was found only for those who were female, White, and more educated. Lastly, the speaker discusses implications for research and practice, as well as directions for future research on educational programs to improve purpose in life among older adults from diverse backgrounds.

